# Molecular Genotyping of Hepatitis A Virus, California, USA, 2017–2018

**DOI:** 10.3201/eid2508.181489

**Published:** 2019-08

**Authors:** William S. Probert, Carlos Gonzalez, Alex Espinosa, Jill K. Hacker

**Affiliations:** California Department of Public Health, Richmond, California, USA

**Keywords:** hepatitis A virus, outbreak, California, genotyping, public health, viruses, United States

## Abstract

We implemented subgenomic and whole-genome sequencing to support the investigation of a large hepatitis A virus outbreak among persons experiencing homelessness, users of illicit drugs, or both in California, USA, during 2017–2018. Genotyping data helped confirm case-patients, track chains of transmission, and monitor the effectiveness of public health control measures.

The United States has seen a resurgence of hepatitis A virus (HAV) infections; several states have reported outbreaks of HAV subgenotype IB, primarily among persons experiencing homelessness and users of illicit drugs (*1*). In California, during November 2016–May 2018, an outbreak of HAV IB infections resulted in 708 case-patients, 465 hospitalizations, and 21 deaths (*1*; https://www.cdph.ca.gov/Programs/CID/DCDC/CDPH%20Document%20Library/Immunization/2016-18CAOutbreakAssociatedDrugUseHomelessness.pdf). To better respond to the surge in hepatitis A cases and facilitate vaccine acquisition and distribution, California declared a public health emergency in October 2017 (*2*). As part of this response, the California Department of Public Health implemented molecular genotyping of HAV to support epidemiologic investigation of suspect cases.

We requested serum samples for symptomatic, HAV IgM–positive case-patients from local public health jurisdictions for genotyping. We amplified a segment of the HAV viral protein 1–amino terminus of 2B (VP1–P2B) genomic region by using nested reverse-transcription PCR and performed sequencing on 160 specimens collected during August 2017–May 2018 (*3*; Appendix). HAV subgenotype classification by VP1–P2B sequence yielded 48 IA-positive, 109 IB-positive, and 3 IIIA-positive specimens (Figure, panel A). We identified 19 unique HAV IA VP1–P2B sequences with an overall average genetic distance of 0.043 nt substitutions per site. Eighteen (37.5%) HAV IA specimens yielded sequences that matched 2 sequences (VRD_521_2016 and RIVM-HAV16–090) previously associated with HAV outbreaks among men who have sex with men (MSM) (*4*,*5*). All 18 case-patients were male, and all but 1 identified as MSM. 

**Figure:**
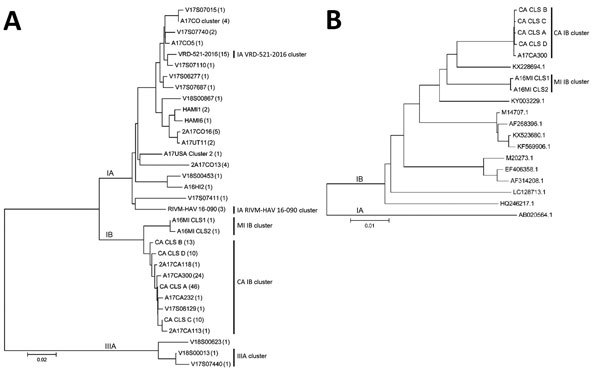
Phylogenetic analysis of HAV sequences from California, USA, and reference sequences. A) Comparison of VP1–P2B sequences obtained for 160 specimens. The number of specimens represented by each VP1–P2B sequence is indicated within parentheses. B) Comparison of nearly complete genome sequences (7,306 nt) for representative subgenotype IB cluster strains with HAV IB strain sequences found in GenBank. The genome sequence (M14707.1) represents the IB reference strain (HM175). A HAV subgenotype IA sequence (AB020564.1) was used as outlier for the analysis of the nearly complete genome sequences. Sequence alignments were performed with ClustalW (http://www.clustal.org), and the dendrograms were generated using the neighbor-joining algorithm and Kimura 2-parameter evolutionary model. Dendrogram branches corresponding to subgenotype lineage are labeled. Identified clusters of HAV are shown to the right of each dendrogram. HAV, hepatitis A virus. Scale bars indicate evolutionary distance.

We identified 11 unique VP1–P2B sequences with an overall average genetic distance of 0.014 nt substitutions per site among the HAV IB specimens. Phylogenetic analysis indicated 2 distinct HAV IB clusters: 1 cluster of 9 closely related sequences (CA IB cluster) identified from 107 California patients, and 1 cluster of 2 specimens matching sequences associated with a concomitant outbreak in Michigan (MI IB cluster) (*1*; Figure). Both case-patients with MI IB strains reported traveling to Michigan during the probable period of exposure. A search of GenBank and Hepatitis A Laboratory Network databases failed to reveal any exact matches to the CA and MI IB outbreak sequences (*6*; https://www.rivm.nl/en/Topics/H/HAVNET). However, Hepatitis A Laboratory Network sequence similarity analysis showed that the CA IB strains were most closely related to strains found in the Middle East and the MI IB strains to strains found in East Africa (data not shown). Three specimens with unique VP1–P2B sequences were classified as subgenotype IIIA, a genotype rarely reported in the United States (*7*). Two of these IIIA sequences (V17S07440 and V18S00013) shared 99.1% and 99.7% sequence identity, respectively, with strains from a 2018 outbreak in Denmark associated with dates imported from Iran (S. Midgley, Statens Serum Institut, Denmark, pers. comm., email, 2018 Aug 22). Neither of those case-patients had traveled internationally or had other known HAV risk factors within their exposure period. However, 1 case-patient had consumed dates from Iran, and the other reported eating dates from a local Middle Eastern grocery store.

We processed selected specimens representing the major IB VP1–P2B sequence variants for whole-genome sequencing (Appendix). Whole-genome sequencing of HAV can provide higher resolution strain typing than sequencing of short subgenomic regions (*8*). We deposited nearly complete genome sequences (7,306 nt) for strains representing the CA and MI outbreaks in GenBank (accession nos. MH577308–14). Phylogenetic comparison with other IB genome sequences in GenBank confirmed that the CA and MI IB outbreak sequences represented distinct clades (Figure, panel B). The CA IB outbreak strains shared 95.5%–95.6% nt and 99.7% aa sequence identity with the HAV IB reference strain, HM175. Similarly, the MI IB outbreak strains shared 95.8% nt and 99.6% aa sequence identity with HM175.

We rapidly implemented genotyping to help guide the public health response to a surge in reported HAV infection cases in California. Paired with epidemiologic data, genotyping information was used to confirm cases, distinguish outbreak-related cases from sporadic cases, track modes and chains of transmission, and identify populations at increased risk for infection. Our study revealed several phylogenetic clusters of HAV. A large cluster of IB strains was confirmed as the primary cause of an outbreak that was chiefly transmitted person-to-person and was associated with risk factors of homelessness and illicit drug use (*1*). Genetically similar strains and risk factors have since been described for outbreaks in other states (*1*). Partly because of these outbreaks, hepatitis A vaccination was recently recommended for persons experiencing homelessness; recommendations for vaccination of users of injection and noninjection drugs were established in 1996 (*9*,*10*). By April 2018, implementation of public health control measures, including educational awareness and targeted vaccination and environmental remediation, reduced the number of reported HAV infection cases to baseline levels in California. 

Limitations of our investigation were the paucity of archival genotyping data from California for strain comparisons and the lack of genotyping capabilities during early stages of the IB outbreak. Sustained public health laboratory capacity for HAV genotyping, along with diligent epidemiologic surveillance, offer the opportunity to detect outbreaks earlier and monitor the effectiveness of prevention and control efforts in California.

Appendix Additional information regarding molecular genotyping of hepatitis A virus, California, 2017–2018.
