# Supplementary material for: Molecular Genotyping of Hepatitis A Virus, California, USA, 2017–2018
Source: Emerg Infect Dis. 2019 Aug;25(8):1594–6. doi: 10.3201/eid2508.181489 (PMC6649344; doi:10.3201/eid2508.181489)
Supplement: Appendix — Additional information regarding molecular genotyping of hepatitis A virus, California, 2017–2018. [file 18-1489-Techapp-s1.pdf]

Article DOI: https://doi.org/10.3201/eid2508.181489

Molecular Genotyping of Hepatitis A Virus, California, USA, 2017–2018

Appendix

Nested RT-PCR Amplification and Sequencing of the VP1-P2B Region of Hepatitis A Virus

Total nucleic acids were extracted and purified from 250 µL of serum using the NucliSENS easyMag system (bioMérieux, Marcy-l'Étoile, France). Nested RT-PCR was performed using the external oligonucleotide primers 2870 and 3381, and the internal oligonucleotide primers 2897 and 3288 (*1*). Twenty microliters of total nucleic acids were reverse transcribed and amplified using the OneStep RT-PCR kit (QIAGEN Inc., Valencia, CA) and the oligonucleotide primers 2870 and 3381 at 300 nM in a total reaction volume of 50 µL. The thermal cycling conditions included a reverse transcription step for 40 min at 50°C, an initial PCR activation step of 15 min at 95°C, followed by a 3-step cycling program of 15 s at 95°C, 20 s at 50°C, and 40 s at 72°C for 45 cycles. A second round of amplification was then performed using the PerfeCTa SYBR Green Fast mix (QuantaBio, Beverly, MA) and included 5 µL of first round amplified product and oligonucleotide primers 2897 and 3288 at 300 nM in a total reaction volume of 50 µL. Thermal cycling parameters for the second round of amplification included an initial PCR activation step of 15 min at 95°C, followed by a 3-step cycling program of 30 s at 95°C, 30 s at 55°C, and 30 s at 72°C for 40 cycles. All amplification reactions were performed with an ABI 9700 thermal cycler (Thermo Fisher Scientific, Waltham, MA). Amplified products were purified using the QIAquick PCR purification kit (QIAGEN inc.) and the purified products subjected to bidirectional dye terminator cycle sequencing using a DNA sequencing service provided by Sequetech Corporation (Mountain View, CA). DNA sequences were assembled, edited, and trimmed to 315 bp with MEGA version 5.05. Phylogenetic analysis was performed in MEGA version 5.05 using ClustalW for sequence alignment and the Neighbor-Joining algorithm and Kimura 2-parameter evolutionary model for dendrogram generation.

Whole-Genome Sequencing of Hepatitis A Virus

Total nucleic acids were extracted and purified as described above and concentrated 5-fold using RNA Clean & Concentrator-5 spin columns (Zymo Research, Irvine, CA). DNA removal was performed using the DNA-free kit (Thermo Fisher Scientific) followed by ribosomal RNA depletion using the NEBNext rRNA depletion kit (New England Biolabs, Ipswich, MA). Libraries were constructed using the NEBNext Ultra II RNA Library Preparation kit (New England Biolabs). Pooled libraries were sequenced using MiSeq Reagent Kit v2 (500-cycles) chemistry on the MiSeq instrument (Illumina, San Diego, CA). Raw FASTQ files were cleaned and contigs generated using the Viral NGS Analysis Platform (CDC, OAMD portal). Final genome assembly and editing was performed with Geneious version 10.0.9 (Biomatters Ltd, Auckland, New Zealand). Genome sequences were trimmed to 7306 nt and phylogenetic analysis was performed in MEGA version 5.05 using ClustalW for sequence alignment and the Neighbor-Joining algorithm and Kimura 2-parameter evolutionary model for dendrogram generation.

Reference

1. Hutin YJF, Pool V, Cramer EH, Nainan OV, Weth J, Williams IT, et al.; National Hepatitis A Investigation Team. A multistate, foodborne outbreak of hepatitis A. N Engl J Med. 1999;340:595–602. PubMed http://dx.doi.org/10.1056/NEJM199902253400802
